# Interpretable prediction of dynamic compressive strength of red sandstone under wetting-drying cycles using ensemble learning

**DOI:** 10.1371/journal.pone.0348902

**Published:** 2026-05-14

**Authors:** Bin Du, Wei Qi, Haibo Bai

**Affiliations:** 1 School of Architectural Construction, Jiangsu Vocational Institute of Architectural Technology, Xuzhou, China; 2 State Key Laboratory of Intelligent Construction and Healthy Operation and Maintenance of Deep Underground Engineering, China University of Mining and Technology, Xuzhou, China; Damghan University, IRAN, ISLAMIC REPUBLIC OF

## Abstract

With geotechnical engineering facing more complex environmental challenges, accurately predicting the mechanical behavior of rocks under dynamic loading becomes essential for maintaining structural safety. In this study, the dynamic compressive strength (DCS) of red sandstone samples under the coupled acidic wetting-drying cycles was investigated, and a dataset comprising 597 test results of experimental samples was obtained. Subsequently, five input variables (strain rate, number of wetting-drying cycles, pH values, uniaxial compressive strength, and P-wave velocity) were used to develop and validate five ensemble learning models: Random Forest (RF), Adaptive Boosting (AdaBoost), Extreme Gradient Boosting (XGBoost), Light Gradient Boosting Machine (LightGBM), and Gradient Boosting Decision Tree (GBDT). The performance of the models was evaluated using four metrics: *R²*, RMSE, MAE, and MAPE, while SHapley Additive exPlanations (SHAP) were employed to interpret feature significance. Among the evaluated models, XGBoost and LightGBM showed similarly strong predictive performance, with XGBoost yielding slightly better overall accuracy under the present dataset conditions. SHAP analysis indicated that strain rate was the most influential factor in DCS prediction, whereas uniaxial compressive strength contributed relatively less. In addition, a graphical user interface (GUI) was developed based on the XGBoost model to facilitate intuitive prediction and visualization of DCS under acidic wetting–drying conditions. Overall, the proposed framework provides an effective and interpretable tool for predicting rock dynamic strength in chemically degraded environments and may support geotechnical stability evaluation and risk reduction.

## Introduction

Red sandstone, a sedimentary rock with moderate to low strength, is extensively found in the red-bed formations of southern China as well as in the arid regions of western China. It commonly features inherent pores and microscopic fractures within its lithological framework, making it particularly vulnerable to physical and chemical deterioration when exposed to environmental stresses and aggressive chemical substances [1–2]. In engineering, red sandstone is commonly encountered in structures such as slopes, tunnels, foundations, and underground excavations. These rock formations are often exposed to repeated wetting and drying cycles under acidic conditions, due to the prolonged contact with atmospheric rainfall, groundwater seepage, industrial wastewater, and acid rain [3]. Under these circumstances, the rock experiences repeated wetting and drying cycles, hydration-dehydration transitions, ion exchange, and the dissolution of cementing materials. These physicochemical reactions gradually weaken the bonds between mineral grains, accelerate pore enlargement, encourage crack merging, and eventually lead to structural disintegration and progressive degradation [4–5]. During acidic wetting-drying cycles, hydrogen ions react with active minerals such as carbonates, iron-aluminum oxides, and silicates, accelerating the dissolution of cementing substances. As a result, the instability of mineral particles is induced, and the integrity of the rock skeleton is damaged [6]. Besides, volume fluctuations caused by swelling-shrinkage behavior and capillary-induced stress under alternating moisture conditions contribute to microcrack initiation and propagation, further deteriorating the rock’s microstructure [7]. The superimposed effects of chemical erosion and mechanical damage have been recognized as fundamental drivers of strength degradation in rocks under acidic wetting-drying environments [8]. These degradation mechanisms also play a significant role in triggering various geological hazards such as landslides, surface erosion, and collapses [9–11].

Existing studies have demonstrated that as the number of wetting-drying cycles increases, the porosity of rock materials progressively increases [12], leading to a consequent reduction in dry density [13]. However, initial compaction or secondary cementation effects may induce scattered P-wave velocity fluctuations, and a progressive attenuation consistently emerges with a continued number of wetting-drying cycles [14]. Meanwhile, mechanical parameters such as elastic modulus [15–16], compressive strength [17–19], tensile strength [20–23], shear strength [24–25], and fracture toughness [26–28] of rock samples exhibit varying degrees of degradation, reflecting a significant decline in structural integrity and load-bearing capacity under repeated wetting-drying cycling. In such environments, rock masses experience prolonged chemical erosion due to acidic substances as well as dynamic disturbances caused by construction operations such as blasting, excavation, and vibration. The interaction of these factors hastens the mechanical deterioration of the red sandstone [29–30]. Under dynamic loading conditions, the dynamic compressive strength (DCS) serves as a key indicator for characterizing the rock’s impact resistance and failure behavior [31–33]. Previous studies have shown that DCS is influenced by multiple factors, including pH levels [34–35], the number of wetting-drying cycles [36], strain rate [37–39], and confining pressure [40–41]. Moreover, DCS is strongly associated with the extent of rock degradation in acidic environments. As structural deterioration progresses, the rock’s impact resistance significantly decreases under high-strain-rate loading, and the synchronized propagation of microcracks rapidly triggers macroscopic failure [42–43].

Traditionally, DCS can be measured through high-strain-rate tests such as the Split Hopkinson Pressure Bar (SHPB). Although these approaches provide high accuracy, their implementation is often hindered by complexity, high costs, and lengthy procedures, making them less feasible for routine engineering applications [44]. In complex multivariate systems, laboratory-based methodologies face inherent constraints in delivering real-time predictive accuracy. With the advancement of computational intelligence, machine learning techniques have emerged as powerful alternatives, offering effective solutions for nonlinear modeling and the analysis of high-dimensional datasets [45–47]. Support Vector Machines (SVM), Random Forests (RF), and Gradient Boosting Decision Trees (GBDT) have shown strong performance across various geotechnical applications for the prediction, analysis and assessment of compressive strength [48–49], elastic modulus [50–51], shear strength [52], rockburst risk [53–54], and microseismic response [55], with high predictive accuracy and generalization capability.

Although machine learning models show great potential in predicting the mechanical behavior of rocks, their performance discrepancies often arise across different algorithms due to variations in modeling strategies, feature encoding methods, and underlying learning principles. These discrepancies manifest most distinctly across key evaluation metrics such as prediction accuracy, robustness, and generalization capability [56]. At present, research on the dynamic strength evolution of rocks under acidic wetting-drying conditions remains limited, and predictive modeling in such intricate environmental conditions is still nascent. To address this gap, a systematic set of experiments involving acidic wetting-drying cycles and dynamic impact compression tests was carried out in this study, generating a robust dataset on the dynamic compressive strength (DCS) of red sandstone. Leveraging this dataset, ensemble tree-based learning models were adopted due to their strong robustness and effectiveness in handling nonlinear relationships in relatively small experimental datasets. Accordingly, five representative ensemble algorithms—RF, AdaBoost, XGBoost, LightGBM, and GBDT—were developed to predict the dynamic compressive strength (DCS) of red sandstone. Finally, the performance of these models was assessed through training and validation procedures, and SHAP values were employed to analyze feature importance and interpret the contribution mechanisms of individual input variables. This study aims to offer theoretical understanding and methodological guidance for the intelligent prediction of rock dynamic strength under cyclic environmental conditions with chemical aggression, thereby advancing the application of data-driven modeling techniques in geotechnical engineering.

## Data preparation and preprocessing

### Data source

All rock-related parameters used in this study were derived from controlled laboratory experiments. Mineralogical analysis via X-ray diffraction (XRD) revealed that the primary constituents are quartz (58.6%), feldspar (21.9%), calcite (9.7%), hematite (6.3%), chlorite (2.2%), and trace minerals (1.3%). The basic physical parameters of the rock mass include a bulk density of 2248 kg/m³, a water absorption rate of 3.24%, a porosity level of 6.58%, and a P-wave velocity of 2513.34 m/s. Specimens for dynamic compressive strength (DCS) testing under acidic wetting-drying conditions were fabricated in accordance with the guidelines established by the International Society for Rock Mechanics (ISRM) [57]. To ensure consistency and reproducibility in the experiments, all specimens were extracted from a single, intact, and macroscopically homogeneous rock block. Cylindrical cores were drilled and then processed into disc-shaped samples with a diameter of 50 mm and a thickness of 30 mm, as shown in Fig 1 in S2 File. Samples exhibiting abnormal P-wave velocity readings were excluded to minimize material heterogeneity.

To simulate the acid-corrosive environments commonly encountered in underground engineering, chemical solutions were prepared using NaCl and KHSO₄, introducing representative ions such as Na ⁺ , K ⁺ , H ⁺ , Cl ⁻ , and SO₄² ⁻ . To accelerate chemical degradation within a limited testing period, the ion concentration was uniformly maintained at 0.1 mol/L. Three chemical conditions were established by adjusting the pH levels: 2.5 (highly acidic), 4.5 (mildly acidic), and 7.0 (neutral, achieved with distilled water). To maintain consistent chemical conditions across all wetting-drying cycles, both pH and ion concentration were adjusted and recalibrated after each cycle. Each wetting-drying cycle comprised two stages: water immersion and thermal dehydration. Specimens were immersed in distilled water at room temperature for 48 hours to achieve complete saturation, then dried in a temperature-controlled oven set at 50 °C for 24 hours. A full cycle was defined as 48 hours of water saturation followed by 24 hours of drying, and the specimens were subjected to different wetting-drying cycle treatments (0, 5, 10, 20, 30, and 40 cycles). The drying temperature was fixed at 50 °C to minimize thermal effects on the rock’s mechanical properties. Once the predetermined number of cycles was completed, the specimens underwent dynamic impact tests via a Split Hopkinson Pressure Bar (SHPB) apparatus. To examine the influence of strain rate on the dynamic mechanical behavior of red sandstone, the air pressure in the gas gun was adjusted to achieve different strain rate conditions, with impact pressures ranging from 0.25 MPa to 0.60 MPa in increments of 0.05 MPa. At each pressure level, at least three replicates were performed to guarantee statistical robustness. Further details of the DCS testing procedure are available in reference [14]. The DCS dataset obtained under acidic wetting-drying cycles is presented in Fig 2 in S2 File.

The rock samples used in this study were obtained from commercially accessible red sandstone material sourced from Linyi City, Shandong Province, China. The sampling location is not located within a protected geological or environmentally restricted area. Therefore, no specific permits or institutional approvals were required for sample collection. The study did not involve protected sites or environmentally sensitive regions.

### Dataset construction

A dataset comprising 597 test results of red sandstone samples subjected to acidic wetting-drying conditions was established for machine learning model development. Five key input variables were selected: strain rate, number of wetting-drying cycles, pH value, uniaxial compressive strength, and P-wave velocity. The DCS was used as the dependent variable. Table 1 shows summary statistics for all parameters. Fig 3 in S2 File shows the frequency distributions of the input and output variables. It can be seen that all variables exhibit relatively uniform distributions with no significant outliers or notable skewness, thereby satisfying the basic statistical assumptions required for machine learning model training.

**Table 1:** Summary statistics of the parameters in the DCS dataset.

Variable	Symbol	Unit	Max	Min	Median	Mean	Std. Dev.	25%	50%	75%
pH value	pH	–	7	2.5	4.5	4.65	1.83	2.5	4.5	7
Number of WD cycles	*n*	–	40	5	20	21.7	13.1	10	20	30
Strain rate	SR	s^-1^	173	41	109	106.89	36.19	75	109	137
Uniaxial compressive strength	UCS	MPa	65.34	43.45	55.23	55.12	5.26	51.34	55.23	59.67
P-wave velocity	V_p_	m/s	2544	1867	2137	2168.3	164	2026	2137	2315
Dynamic compressive strength	DCS	MPa	213.8	23.2	125	125.76	38	97.4	125	152.8

### Correlation analysis of model parameters

In machine learning, high correlations among input features can lead to multicollinearity, potentially undermining model performance and interpretability. Therefore, correlation coefficients above 0.8 are commonly regarded as indicators requiring careful examination rather than strict exclusion criteria. In physics-informed problems, strongly correlated variables may still represent distinct mechanistic information and should be evaluated in conjunction with their physical meaning [58]. Adhering to this principle, Pearson’s correlation coefficient was used to assess the linear associations among the selected input features. Fig 4 in S2 File displays the obtained correlation matrix. Within this matrix, the intensity and color variation of each cell reflect the magnitude and direction of the correlations: positive coefficients represent direct associations, negative coefficients suggest inverse relationships, and values closer to ±1 indicate stronger linear dependencies.

As shown in Fig 4 in S2 File, DCS exhibits positive correlations with strain rate (SR), pH, uniaxial compressive strength (UCS), and P-wave velocity (V_p_), and a negative correlation with the number of wetting-drying cycles (*n)*. The strongest positive correlations are found between DCS and both SR and V_p_, with correlation coefficients reaching 0.65. In contrast, a strong negative correlation of −0.86 is observed between *n* and V_p_. In the present study, this relationship is physically meaningful rather than merely indicative of redundant information. Specifically, *n* represents the imposed degradation history through wetting–drying cycles, whereas V_p_ reflects the resulting internal structural response associated with pore evolution, microcrack propagation, and stiffness degradation. Their correlation therefore captures the progressive linkage between environmental deterioration and material integrity. Accordingly, correlation coefficients above 0.8 were treated in this study as warning indicators requiring physical interpretation rather than automatic exclusion criteria. Moreover, the tree-based ensemble models employed here are generally less sensitive to multicollinearity than coefficient-based methods. Retaining both variables therefore preserves mechanistically relevant information and is not expected to substantially compromise predictive modeling [59].

## Ensemble learning algorithms

### RF

Random Forest (RF) is a widely used ensemble learning algorithm that improves prediction performance by constructing multiple decision trees and aggregating their outputs. To minimize overfitting and improve model robustness, two fundamental techniques are used in RF: bootstrap aggregation (commonly known as bagging) and the random selection of features during tree construction. Fig 5 in S2 File presents a schematic diagram of the training process of the random forest regression model. In this process, each decision tree is constructed using a bootstrapped subset of the initial dataset (i.e., sampled with replacement), and at every node, an optimal split is determined by evaluating a randomly chosen subset of features [60]. This procedure enhances the diversity of the model and minimizes the interdependence among decision trees. The RF method integrates several weak learners (each being a single decision tree) to form a robust predictive model. In the context of regression problems, the final prediction is derived by computing the average of all individual tree outputs. Mathematically, the overall predicted value generated by the RF model can be represented as:


$$\hat{y}=\frac{\text{1}}{T}\sum_{t=\text{1}}^{T}f_{t}(x)$$ (1)

Where *T* denotes the total number of decision trees in the ensemble, $f_{t}(x)\;$ represents the prediction output of the 𝑡-th decision tree, and *x* is the input feature vector.

### AdaBoost

AdaBoost is a boosting-based ensemble learning algorithm, and its prediction accuracy is improved by sequentially training a series of weak learners—typically shallow decision trees—and increasing focus on misclassified instances. Fig 6 in S2 File presents the flowchart of the AdaBoost algorithm. During each iteration, the weights assigned to samples are adjusted according to the errors made by the preceding weak learner, such that greater emphasis is placed on incorrectly predicted samples. This iterative reweighting guides subsequent learners to correct previous mistakes, thereby enhancing overall model performance. The final prediction is generated by aggregating the outputs of all weak learners through a weighted sum, thereby effectively converting multiple weak learners into a strong learner [61]. For classification or regression tasks, the AdaBoost prediction can be expressed as follows:


$$\text{F}(x)=\text{sign}\left(\sum_{m=\text{1}}^{M}\alpha_{m}h_{m}(x)\right)$$ (2)

where $h_{m}(x)$ denotes the *m*-th weak classifier, $\alpha_{m}=\frac{\text{1}}{\text{2}}\text{ln}\left(\frac{\text{1}-\epsilon_{m}}{\epsilon_{m}}\right)$ is the weight assigned to the 𝑚-th weak classifier, and $\epsilon_{m}$ is the classification error of the 𝑚-th iteration.

### XGBoost

XGBoost is an ensemble learning algorithm rooted in gradient boosting, offering significant enhancements compared to conventional approaches. As illustrated in Fig 7 in S2 File, the model incorporates second-order derivatives in loss function optimization, introduces a regularization term to manage the model complexity, and supports parallelized and distributed computation, leading to enhanced prediction accuracy and computational efficiency [62]. In XGBoost, decision trees are constructed in a sequential manner, where each subsequent tree focuses on reducing the residuals left by the previous ensemble by minimizing the cumulative loss. To prevent overfitting, a regularization component is incorporated to penalize excessive model complexity. Furthermore, XGBoost enhances reliability and scalability through features like native support for missing data, column-wise sub-sampling, and highly efficient parallel processing, establishing it as one of the most versatile and powerful gradient boosting frameworks. The objective function of XGBoost is defined as:


$$\text{Obj}=\sum_{i=\text{1}}^{n}l\left(y_{i},{\hat{y}}_{i}\right)+\sum_{t=\text{1}}^{T}\Omega \left(f_{t}\right)$$ (3)

Where *l* is the loss function measuring the difference between the true and predicted values, $\Omega \left(f_{t}\right)$ is the regularization term for the *t*-th tree, T the *t*otal number of trees, and $f_{t}$ represents the prediction function of the *t*-th tree.

### LightGBM

LightGBM is a gradient boosting framework optimized for large-scale datasets and high-dimensional feature spaces. Fig 8 in S2 File shows the flowchart of the LightGBM algorithm. In contrast to XGBoost, LightGBM achieves superior training efficiency and competitive prediction accuracy through two core techniques: a histogram-based decision tree algorithm and a leaf-wise growth strategy [63]. The histogram-based approach discretizes continuous features into fixed bins, substantially reducing memory consumption and computational overhead. The leaf-wise growth strategy expands trees by selecting the leaf node with the maximum loss reduction, following the gradient descent direction, thereby facilitating a more adaptive and efficient model training process. LightGBM also supports GPU acceleration and parallel learning, demonstrating strong performance on large-scale, high-dimensional, and sparse datasets. Its objective function is similar to that of XGBoost and can be formulated as:


$$\text{Obj}=\sum_{i=\text{1}}^{n}l\left(y_{i},{\hat{y}}_{i}\right)+\lambda \sum_{t=\text{1}}^{T}\left({\left\|\omega_{t}\right\|}^{\text{2}}\right)$$ (4)

where *l* denotes the loss function, $\omega_{t}$ is the weight of the *t*-th leaf node, and λ is the regularization coefficien*t* controlling model complexity.

### GBDT

GBDT, a well-known boosting algorithm, adopts an additive model by sequentially integrating a series of weak learners, most commonly decision regression trees. As shown in Fig 9 in S2 File, during each iteration, GBDT fits a new tree to the negative gradient of the loss function (i.e., the residuals) of the current model, thereby gradually minimizing the overall prediction error [64]. GBDT has been widely used for both regression and classification tasks due to its strong nonlinear fitting capability and built-in feature selection. Owing to its remarkable versatility and proven efficacy, GBDT has solidified its position as a cornerstone technique within numerous ensemble learning methods. The prediction output of a GBDT model can be expressed as:


$$F_{M}(x)=\sum_{m=\text{1}}^{M}\gamma_{m}h_{m}(x)$$ (5)

Where $F_{M}(x)$ denotes the final prediction result after *M* iterations, $h_{m}(x)\;$represents the *m*-th regression tree, and $\gamma_{m}$ is the learning rate that controls the contribution of each individual tree to the overall model.

## Model implementation and results analysis

### Model training and testing workflow

The construction of machine learning models to forecast the dynamic compressive strength (DCS) of red sandstone was carried out according to the following procedure:

(1) Data Collection

A dataset comprising 597 test results of experimental instances was assembled, with each sample characterized by five key input features: SR, *n*, pH, UCS, and V_p_. The corresponding DCS served as the output variable.

(2) Data Processing

Before model development, all variables underwent data cleaning and were normalized through Min-Max Scaling to transform values into the interval [0, 1], effectively mitigating discrepancies among features [65]. The processed dataset was then randomly partitioned into a training subset (478 samples, accounting for 80% of the total) and a testing subset (119 samples, 20%), ensuring an impartial assessment of model performance.

(3) Model Training

To establish nonlinear relationships between input features and the target variable, five ensemble learning algorithms were utilized. During the training phase, a 10-fold cross-validation approach was adopted to improve model robustness and generalization performance and facilitate the hyperparameter optimization.

(4) Hyperparameter Optimization

A grid search method integrated with 10-fold cross-validation was applied to systematically evaluate predefined hyperparameter configurations and determine the best settings for each model [66]. For each candidate hyperparameter combination, the mean *R*² value across the 10 folds was used as the optimization criterion, and the combination yielding the highest average *R*² was selected as the final optimal parameter set. This process was designed to achieve optimal prediction accuracy and reduce the risk of overfitting. Hyperparameter ranges were selected according to commonly reported intervals for tree-based ensemble models to ensure exploration across both underfitting and overfitting regimes. The search ranges and selected hyperparameters are summarized in Table 2.

**Table 2:** Optimal Hyperparameters for Ensemble Learning Models.

Model	Hyperparameter	Search Range	Optimal Value
RF	max_depth	5, 10, 15, 20	10
	min_samples_leaf	1, 2, 4, 6	2
	min_samples_split	2, 4, 6, 8	2
	n_estimators	100, 200, 300, 500	200
Adaboost	learning_rate	0.01, 0.05, 0.1, 0.5, 1.0	1.0
	loss	linear, square, exponential	square
	*n*_estimators	50, 100, 200, 300	200
XGBoost	colsample_bytree	0.6, 0.7, 0.8, 0.9, 1.0	0.8
	learning_rate	0.01, 0.05, 0.1, 0.2	0.1
	max_depth	3, 4, 5, 6	3
	n_estimators	50, 100, 200, 300	100
	subsample	0.6, 0.7, 0.8, 0.9, 1.0	0.8
LightGBM	colsample_bytree	0.6, 0.7, 0.8, 0.9, 1.0	0.8
	learning_rate	0.01, 0.05, 0.1, 0.2	0.1
	max_depth	3, 4, 5, 6	3
	n_estimators	100, 200, 300, 400	200
	num_leaves	10, 20, 31, 63	20
	subsample	0.6, 0.7, 0.8, 0.9, 1.0	0.8
GBDT	learning_rate	0.01, 0.05, 0.1, 0.2	0.1
	max_depth	3, 4, 5, 6	3
	*n*_estimators	50, 100, 200, 300	100
	subsample	0.6, 0.7, 0.8, 0.9, 1.0	0.8

(5) Model Evaluation

Model performance was assessed on the independent testing dataset using four evaluation metrics: coefficient of determination (*R²*), mean absolute error (MAE), root mean square error (RMSE), and mean absolute percentage error (MAPE). Table 3 shows the definitions of these metrics. In addition, cross-validation results were examined to evaluate model stability and generalization across different data subsets.

**Table 3:** Evaluation Metrics for Prediction Models.

Metric	Formula	Ideal Value	Description	Evaluation Criteria
*R* ^*2*^	$R^{\text{2}}=\text{1}-\frac{\sum_{i=\text{1}}^{n}{\left(y_{i}-{\hat{y}}_{i}\right)}^{\text{2}}}{\sum_{i=\text{1}}^{n}{\left(y_{i}-\overset{―}{y}\right)}^{\text{2}}}$	1	It measures the model’s ability to fit the variability of observed data.	A value closer to 1 indicates a better fitting performance and superior predictive capability.
MAE	$\text{MAE}=\frac{\text{1}}{n}\sum_{i=\text{1}}^{n}\left|y_{i}-{\hat{y}}_{i}\right|$	0	It represents the average absolute deviation between the predicted and actual values.	A smaller value indicates more accurate predictions, lower errors, and better model performance.
RMSE	$\text{RMSE}=\sqrt{\frac{\text{1}}{n}\sum_{i=\text{1}}^{n}{\left(y_{i}-{\hat{y}}_{i}\right)}^{\text{2}}}$	0	It reflects the variability of the prediction errors.	A smaller value indicates lower overall error variability, implying higher model stability and greater prediction accuracy.
MAPE	$\text{MAPE}=\frac{\text{1}}{n}\sum_{i=\text{1}}^{n}\left|\frac{y_{i}-{\hat{y}}_{i}}{y_{i}}\right|\times \text{1}\text{0}\text{0}\%$	0	It measures the proportion of error between the predicted and actual values.	A smaller value indicates lower relative error, reflecting higher relative accuracy of the model’s predictions.

(6) Model Interpretation

To improve the model interpretability, feature importance analysis and SHAP values were employed. These methods quantified the contribution of each input variable to the model’s predictions, offering insights into the internal decision-making processes of the algorithms.

Fig 10 in S2 File presents the overall technical framework of this study.

### Evaluation of model predictive performance

Figs 11 and 12 in S2 File display the comparison between predicted and actual values, as well as the corresponding percentage absolute error distributions for the five machine learning models. All models demonstrate strong predictive performance, with the majority of predictions falling within a ± 10% error range. These findings indicate that the ensemble learning algorithms employed are effective in capturing the underlying relationships between input features and DCS of red sandstone under acidic wetting-drying cycles.

Table 4 presents the performance metrics of the five machine learning models on testing sets. For the testing set, all models achieve satisfactory predictive performance, with *R*² values greater than 0.90, confirming good generalization ability. Among them, LightGBM and GBDT achieve slightly better overall predictive accuracy, reflected by lower RMSE and MAPE values, while RF also maintains stable performance across all evaluation metrics. XGBoost shows comparable predictive capability but does not consistently outperform the other models across all indicators. In contrast, AdaBoost yields comparatively higher prediction errors, suggesting limited suitability for the present prediction task. Furthermore, the a-10 and a-20 indicators indicate that most predictions fall within acceptable engineering error ranges, further confirming the reliability of the developed models.

**Table 4:** Performance evaluation metrics of different machine learning models.

Model	*R* ^*2*^		MAE		RMSE		MAPE		a-10 (%)	a-20 (%)	Total rank
	Value	Rank	Value	Rank	Value	Rank	Value	Rank			
RF	0.9229	2	8.3836	2	10.7703	2	7.2451	2	77.5	96.67	8
Adaboost	0.9005	1	9.8454	1	12.2346	1	8.6768	1	72.5	91.67	4
XGBoost	0.9413	5	7.5906	4	9.3968	5	6.6468	4	77.5	96.67	18
LightGBM	0.9390	4	7.5894	5	9.5828	4	6.5422	5	80.83	98.33	18
GBDT	0.9358	3	7.7642	3	9.8321	3	6.8457	3	79.17	96.67	12

To quantitatively compare the overall performance of the models, a scoring framework was implemented based on the approach proposed by Zorlu et al. [67]. Specifically, for each evaluation metric, the best-performing model was assigned 5 points and the worst-performing model was given 1 point, with intermediate scores allocated accordingly. Total scores were calculated by aggregating the values across the four evaluation metrics to reflect each model’s comprehensive performance. Table 4 presents the scoring results of the evaluated models based on testing dataset performance. Both XGBoost and LightGBM achieved the highest total scores, indicating superior overall predictive capability. GBDT ranked next with moderate performance, followed by Random Forest. AdaBoost obtained the lowest score, suggesting comparatively limited suitability for the present prediction task.

The observed performance differences among the models can be attributed to their underlying learning mechanisms and their adaptability to the characteristics of the present dataset. Gradient boosting–based algorithms such as XGBoost and LightGBM iteratively optimize prediction residuals and are capable of effectively capturing complex nonlinear relationships and feature interactions inherent in rock mechanical behavior. In particular, these models benefit from efficient tree-splitting strategies and regularization mechanisms, which enhance generalization performance when dealing with tabular datasets of moderate size. GBDT demonstrates comparable predictive capability but lacks some of the optimization strategies incorporated in more advanced boosting frameworks, resulting in slightly reduced accuracy. Random Forest, based on bagging aggregation, provides stable predictions and strong robustness but may be less effective in modeling subtle nonlinear dependencies compared with boosting-based approaches. In contrast, AdaBoost relies on sequential reweighting of samples and is more sensitive to noise and local fluctuations in experimental data, which may reduce prediction stability when modeling heterogeneous damage evolution processes in rocks [68–69]. These algorithmic characteristics provide a reasonable explanation for the performance ranking observed in Table 4.

To further examine the robustness of the selected model, the XGBoost model was additionally evaluated using 10-fold cross-validation. The results showed consistent predictive performance across folds, with an average *R*² of 0.9377 ± 0.0119, MAE of 7.38 ± 0.71 MPa, RMSE of 9.37 ± 0.83 MPa, and MAPE of 6.73 ± 1.03%. The relatively small standard deviations indicate that the predictive performance was stable across different folds and did not depend on a particularly favorable data split.

### Feature importance analysis based on the SHAP interpretability method

SHAP establishes a rigorous mathematical framework for model interpretability in machine learning by quantifying both the magnitude and direction of each input feature’s contribution to the predicted output [70]. In the context of rock strength prediction, the SHAP framework enables transparent analysis of the influence of key variables (such as strain rate, pH value, and the number of wetting-drying cycles) on predictive outcomes. Beyond delineating individual parametric influences, SHAP also captures nonlinear interactions and assesses the model compliance with fundamental rock mechanical principles. Visualization methods, including feature importance rankings, dependence plots, and interaction diagrams, further enhance model interpretability and strengthen the engineering credibility of machine learning applications in geotechnical engineering.

#### Global interpretability.

Fig 13 in S2 File presents the feature importance analysis based on SHAP values obtained from the XGBoost model. The x-axis shows the mean absolute SHAP value for each input feature, representing its average contribution to the model’s output. By using absolute values, the analysis highlights the magnitude of the influence of input features without regard to their directionality [71]. Features along the y-axis are ordered by their significance in descending sequence, where the length of each bar reflects the relative influence on DCS prediction. The results indicate that SR has the greatest impact, followed by pH, V_p_, and *n*. In contrast, UCS exhibits the least contribution, as evidenced by its minimal SHAP value. From a mechanistic perspective, SR is a key parameter governing rock deformation under dynamic loading conditions. When strain rates increase, the propagation of microcracks is constrained and energy accumulation occurs rapidly, resulting in sudden failure accompanied by higher material strength. At higher strain rates, crack propagation is suppressed and energy accumulates rapidly, often leading to the elevated strength of the materials and the abrupt failure [44]. This dynamic effect alters both the failure mode and the apparent strength of the material. In contrast, UCS reflects static loading conditions and exhibits limited sensitivity to the coupled effects of dynamic loading and chemical degradation. Therefore, UCS has the least influence on the outcome. It should be noted that SHAP values quantify the relative contribution of input variables to model predictions under the current dataset conditions and variable system. Therefore, the SHAP-based importance ranking should not be directly interpreted as an absolute ranking of physical importance in a rock mechanics sense.

Fig 14 in S2 File presents the SHAP summary plot for all input features. It offers a comprehensive visualization of both the magnitude and direction of each feature’s influence on the predicted DCS. The SHAP values are displayed in the x-axis, quantifying different contributions of input features to the model output across all samples. The input features are arranged on the y-axis and ordered by importance. Each point corresponds to an individual sample, where the color reflects the associated feature value (red denotes higher values, while blue indicates lower values) [72]. This plot captures not only the average importance of each feature but also illustrates the distribution and directional effect of feature values across the dataset. The SHAP summary plot indicates that SR, pH, V_p_, and UCS have a predominantly positive influence on DCS, suggesting that higher values of these variables are generally associated with increased dynamic strength. In contrast, *n* exhibits a negative relationship with DCS, indicating that an increase in cycle number tends to reduce the rock’s dynamic strength.

#### Local interpretation after global SHAP.

Global SHAP analysis reflects the average importance of variables across the entire sample space. However, dynamic rock strength is jointly governed by the coupled effects of loading rate, damage evolution, and structural integrity, meaning that the same variable may exert different influences under different conditions. To verify whether the model maintains physical consistency at the individual-sample level and to reveal dominant mechanisms across different strength regimes, SHAP waterfall plots were employed for local interpretation (Fig 15 in S2 File) [73–74].

In the waterfall plot, the baseline value E[f(x)] (125.413 MPa in this study) serves as the starting point. Red bars indicate positive contributions that increase the prediction, whereas blue bars represent negative contributions that decrease it. The length of each bar reflects the magnitude of the feature’s contribution to the final prediction f(x). Based on the distribution of DCS values in the testing dataset, three representative samples were selected according to strength level: a low-strength sample representing the damage-dominated regime, a medium-strength sample representing a competitive equilibrium among multiple factors, and a high-strength sample representing the upper-bound regime controlled by the synergy between strain-rate strengthening and structural integrity. This three-scale selection covers the principal strength intervals and enables comparison of model interpretability across different mechanical states.

(1) Low Dynamic Strength Sample

For the low-strength representative sample, Fig 15(a) in S2 File shows that the true dynamic compressive strength is 41.80 MPa, while the model predicts 55.01 MPa, indicating a moderate overestimation of approximately +13.21 MPa. The SHAP waterfall plot shows that the prediction is substantially reduced from the baseline value of 125.413 MPa. The extremely low strain rate (SR = 0.030) provides the dominant negative contribution (approximately −41 MPa), demonstrating that the model correctly identifies the insufficient dynamic strengthening effect under low loading-rate conditions. Meanwhile, the high number of wetting–drying cycles (*n* = 1.0) and the low longitudinal wave velocity (V_p_ = 0.083) further indicate accumulated structural damage and reduced material integrity, jointly driving the predicted strength downward.

Although the model successfully captures the primary weakening mechanisms, the prediction remains slightly higher than the measured value. This discrepancy suggests that the actual degradation level in extremely weak samples may exceed the average trend learned by the model. Such deviations are common in data-driven models, particularly when low-strength samples are relatively limited, causing predictions to regress toward the dataset mean. Nevertheless, the direction and dominance of feature contributions, as reflected in Fig 15(a) in S2 File, remain consistent with established rock dynamic behavior, confirming the physical interpretability of the model in the low-strength regime.

(2) Medium Dynamic Strength Sample

The medium-strength sample, illustrated in Fig 15(b) in S2 File, exhibits a true dynamic compressive strength of 130.90 MPa, whereas the predicted value is 117.85 MPa, corresponding to a slight underestimation of approximately −13.05 MPa. The predicted value lies close to the baseline level, indicating a competitive balance among multiple influencing factors. According to the SHAP interpretation, a relatively low strain rate (SR = 0.091) still imposes a strong negative contribution (about −35 MPa), acting as the primary constraint on strength enhancement. In contrast, higher static compressive strength (UCS = 0.726), moderate wave velocity (V_p_ = 0.526), and a smaller number of wetting–drying cycles (*n* = 0.143) collectively provide positive compensations, bringing the prediction back toward the baseline.

The slight underestimation may arise from local microstructural advantages or experimental variability that result in higher measured strength than the statistical trend captured by the model. Importantly, this sample represents a transitional state between damage-dominated and rate–structure coupled control regimes, illustrating the model’s capability to recognize the dynamic balance among competing mechanisms within the medium-strength range.

(3) High Dynamic Strength Sample

A different pattern is observed for the high-strength case (Fig 15(c) in S2 File). The true dynamic compressive strength reaches 213.80 MPa, while the predicted value is 208.68 MPa, yielding a small error of only −5.12 MPa, which demonstrates excellent predictive accuracy. The local SHAP analysis indicates that the high strain rate (SR = 0.864) provides the most significant positive contribution (approximately +34.8 MPa), serving as the decisive factor elevating the prediction far above the baseline value. Additionally, the high longitudinal wave velocity (V_p_ = 0.966) contributes roughly +20 MPa, reflecting high structural integrity that enables strong dynamic strengthening under rapid loading conditions.

Compared with the low- and medium-strength cases, positive contributions in this sample exhibit clear synergistic effects, suggesting that when both loading rate and structural integrity reach high levels, the model can reliably capture the dynamic strengthening mechanism, resulting in markedly reduced prediction error. The relatively small or slightly negative contribution of UCS does not imply reduced physical importance but likely reflects partial redundancy with V_p_ or SR, whereby the model compresses its marginal contribution during local attribution. Overall, this case validates, at the individual-sample level, the global SHAP conclusion that strain rate is the primary driving factor governing dynamic strength enhancement.

In summary, the local interpretations across different strength regimes, summarized in Fig 15(a)–(c) in S2 File, show strong consistency with the global SHAP importance ranking [75–76]. This agreement demonstrates that the proposed model not only achieves high predictive accuracy but also reveals physically meaningful mechanisms governing the evolution of dynamic compressive strength at the individual-sample scale.

#### Feature dependence analysis.

Fig 16 in S2 File illustrates the SHAP dependence plots for individual features. The x-axis represents the actual feature values of the samples, while the y-axis shows the corresponding SHAP values [77]. These SHAP values can be used to quantify each feature’s contribution to the predicted DCS.

(1) A strong positive correlation is observed between SR and DCS, as shown in Fig 16(a) in S2 File. As SR increases, the corresponding SHAP values also increase in a near-linear trend, indicating a consistently positive influence on the model’s prediction. This relationship indicates that under high strain rate conditions, the dynamic bearing capacity of the rock can be enhanced, which is consistent with the known mechanism of increased strength resulting from crack inhibition and inertial confinement during the impact loading process.
(2) As shown in Fig 16(b) in S2 File, the relationship between pH and DCS displays a distinct nonlinear trend. Under highly acidic conditions, SHAP values are mainly negative, indicating that acidic conditions weaken the DCS of red sandstone. When pH moves toward neutral levels, SHAP values transition to positive, suggesting that neutral conditions are more favorable for maintaining rock strength. This pattern reflects that acidic solutions can severely damage the microstructure of rock due to the dissolution of the cement matrix and the separation of minerals, leading to the formation of microcracks and a decline in overall mechanical strength.
(3) As shown in Fig 16(c) in S2 File, a strong positive correlation is observed between V_p_ and DCS. Higher V_p_ values are associated with larger SHAP values, indicating a greater positive influence on model predictions. Physically, V_p_ serves as a sensitive indicator of rock density, structural integrity, and elastic stiffness. A higher V_p_ typically reflects a more compact and homogeneous rock matrix with fewer internal defects, thereby improving the material’s ability to withstand compressive failure under dynamic stress conditions.
(4) Fig 16(d) in S2 File shows a negative correlation between *n* and SHAP values associated with DCS. As *n* increases, SHAP values consistently decrease, indicating that repeated wetting-drying cycles significantly reduce the model’s prediction of rock strength. This pattern aligns with the known degradation mechanisms, including cementitious matrix breakdown, pore enlargement, and cumulative microcrack propagation. The mentioned degradation behaviors of rock mass progressively weaken its internal structure under cyclic wetting-drying conditions.
(5) Fig 17(e) in S2 File indicates a positive correlation between UCS and SHAP values. As UCS increases, SHAP values also rise, suggesting that specimens exhibiting greater UCS tend to be predicted with higher DCS. As a macroscopic measure of rock strength, UCS is typically linked to microstructural compactness, mineral composition, and the degree of cementation—all of which contribute to enhanced dynamic mechanical performance.

### Development of the GUI

In this study, a predictive system for DCS of rock was developed using the Python programming language based on a three-tier architecture, as shown in Fig 17 in S1 File. The system architecture consists of three main components: a user interface layer developed with the PyQt5 framework, a business logic layer that incorporates an enhanced XGBoost model, and a data modeling layer tasked with feature normalization and preprocessing. A modular design approach was adopted to decouple functionalities across layers, thereby improving system scalability, maintainability, and potential for future extension in practical geotechnical applications.

The core features of the system include: (1) the construction of a XGBoost-based prediction model with optimized hyperparameters, incorporating Min-Max scaling for feature normalization; (2) the development of an interactive graphical user interface (GUI) that supports parameter input, result display, and model evaluation; (3) the implementation of a complete workflow encompassing input validation, data preprocessing, model inference, and output visualization.

By entering five key rock parameters, users can obtain real-time predictions of DCS along with corresponding evaluation metrics. The system overcomes major drawbacks of conventional rock mechanics testing—such as prolonged experimental durations and low efficiency—by providing an intelligent, efficient, and intuitive solution tailored for geotechnical engineering applications. This tool shows significant promise for real-world implementation in large-scale infrastructure developments, such as road and railway tunnels, as well as hydropower facilities. Moreover, with the continued accumulation of sample data, the system enables incremental learning, facilitating continuous model improvement and supporting the wider digitalization of the geotechnical field.

## Limitations and future research

Although the proposed ensemble learning framework achieved satisfactory performance in predicting the dynamic compressive strength of red sandstone under wetting–drying cycles, several limitations should be acknowledged.

First, the present model was developed using data generated under controlled laboratory conditions. This design was beneficial for isolating the effects of key variables and improving model identifiability, but it also limits direct extrapolation to more complex geological settings. In natural underground environments, hydrochemical conditions, stress states, exposure durations, and structural heterogeneity are often much more complicated than those represented in laboratory experiments. As a result, the variable relationships identified in this study may shift when extended to field-scale applications.

Second, although both global and local SHAP analyses were incorporated to improve model transparency, the interpretability results remain constrained by the current data structure and variable system [78]. In dynamic rock mechanics, strength evolution is inherently governed by coupled processes, and the direction as well as the magnitude of feature contributions may vary across samples and conditions. Therefore, the current interpretability analysis should be regarded as a meaningful description of model behavior under the present experimental framework, rather than a complete reconstruction of all underlying physical mechanisms. Future work should integrate additional microstructural characterization, such as pore evolution, crack development, and mineralogical alteration, to provide stronger support for mechanism-based interpretation.

In addition, the comparative performance of the ensemble models should be understood as a dataset-dependent evaluation rather than a universal ranking of algorithm superiority. The close performance of several models suggests that predictive behavior is influenced not only by algorithm design, but also by dataset size, feature composition, and validation strategy. Broader comparisons using larger datasets and more diverse input configurations would therefore be valuable for assessing the stability of the present conclusions.

Based on these limitations, several directions for future research can be identified. First, the dataset should be expanded to include different rock types, wider hydrochemical conditions, and more diverse loading paths in order to improve model generalizability. Second, microstructural and mineralogical descriptors should be combined with conventional macroscopic parameters to strengthen the representation of degradation processes. Third, external validation using field monitoring data or engineering case data would help bridge the gap between laboratory-based modeling and practical applications. Finally, future studies may further develop cross-scale interpretability frameworks that connect global feature importance, local sample-level explanation, and independent experimental evidence, thereby improving both the physical credibility and engineering applicability of predictive models for dynamic rock strength.

## Conclusions

Conventional lab-based techniques for assessing rock DCS under intricate environmental conditions typically require substantial time and resources, which restricts their effectiveness in engineering scenarios with tight deadlines. To this end, an ensemble learning-based prediction framework for DCS was proposed, offering an efficient and reliable alternative for geotechnical engineering applications. Five ensemble learning models were developed and systematically evaluated using experimental data from red sandstone specimens. To enhance interpretability and reduce the black-box nature of complex models, SHAP-based explainable machine learning techniques were employed to quantify the influence of each input feature on DCS predictions. In addition, a user-friendly GUI was built upon the best-performing XGBoost model, enabling real-time strength prediction with practical relevance for field applications. The main conclusions of this study are as follows:

(1) An ensemble learning-based prediction framework for estimating DCS of red sandstone was developed, and five key influencing parameters were systematically incorporated. A dataset containing 597 test results of red sandstone samples was used to train and validate the performance of five ensemble learning models: RF, AdaBoost, XGBoost, LightGBM, and GBDT. The results confirm that ensemble learning methods offer strong predictive accuracy and robustness for modeling rock mechanical behavior under chemically aggressive and dynamically loaded conditions.
(2) Among the five evaluated models, RF, LightGBM, GBDT, and XGBoost all showed satisfactory predictive performance, with LightGBM and XGBoost achieving slightly better overall results under the present dataset conditions. These findings suggest that tree-based ensemble learning models provide an effective framework for predicting the dynamic compressive strength of red sandstone under coupled acidic wetting–drying conditions, whereas AdaBoost showed comparatively lower predictive performance.
(3) Global and local SHAP analyses consistently indicated that SR was the primary factor controlling DCS, whereas UCS contributed relatively less under the present dataset conditions. The results further showed nonlinear effects of SR, pH, and V_p_, a generally positive role of UCS, and a negative influence of wetting–drying cycles. The local interpretation of representative low-, medium-, and high-strength samples confirmed that these contributions were sample-dependent and reflected meaningful interactions among loading conditions, degradation processes, and rock integrity.
(4) An optimized XGBoost model was used to develop a graphical user interface (GUI) for predicting the durability of red sandstone under acidic wetting-drying cycles. The GUI features a streamlined, user-friendly design that enables efficient strength assessments under acidic wetting-drying conditions, offering practical utility for geotechnical engineering applications.

## Supporting information

S1 File Minimal data set. (XLSX)

S2 File Fig 1. Red sandstone specimens used for dynamic impact testing. Fig 2. DCS dataset of red sandstone under acidic wetting-drying conditions. Fig 3. Frequency distribution of input and output variables in the dataset. Fig 4. Pearson correlation matrix of input features and DCS. Fig 5. Schematic diagram of the RF algorithm principle. Fig 6. Flowchart of the AdaBoost algorithm. Fig 7. Schematic diagram of the XGBoost model. Fig 8. LightGBM algorithm flow chart. Fig 9. Flowchart of the GBDT algorithm. Fig 10. Overall framework of the dynamic strength prediction model for red sandstone after wetting-drying cycles. Fig 11. Predicted results of DCS by different ensemble learning models. (a) RF (b) Adaboost (c) XGBoost (d) LightGBM (e) GBDT. Fig 12. Percentage absolute error distribution of DCS predictions for different models. (a) RF (b) Adaboost (c) XGBoost (d) LightGBM (e) GBDT. Fig 13. Feature importance of input variables based on SHAP values. Fig 14. SHAP summary plot of input variables. Fig 15. SHAP waterfall plots for local interpretation of representative low-, medium-, and high-strength samples. (a) low-strength sample (b) medium-strength sample (c) high-strength sample. Fig 16 Analysis of the influence of each input features on the target variable. (a) SR (b) pH (c) Vp (d) n (e) UCS. Fig 17 GUI for DCS prediction using the XGBoost model.
(RAR)
